# *BIRC3/CAV1* co-expression drives GBM aggressiveness as a prognostic signature and therapeutic vulnerability

**DOI:** 10.1038/s41420-026-03112-z

**Published:** 2026-04-14

**Authors:** Sara Franceschi, Mariangela Morelli, Francesca Lessi, Francesca Di Lorenzo, Paolo Aretini, Aldo Pastore, Andrea Marranci, Carlo Gambacciani, Francesco Pieri, Federico Villanacci, Nicola Montemurro, Manuel Giacomarra, Michele Menicagli, Gianmarco Ferri, Francesco Pasqualetti, Marco Krengli, Marc Sanson, Alberto Picca, Anna Luisa Di Stefano, Orazio Santo Santonocito, Chiara Maria Mazzanti

**Affiliations:** 1 Fondazione Pisana per la Scienza, Pisa, Italy; 2 Scuola Normale Superiore, Pisa, Italy; 3 Department of Neurosurgery, Azienda Ospedaliera Toscana Nord-ovest, Livorno, Italy; 4 Department of Neurosurgery, Azienda Ospedaliera Universitaria Pisana, Pisa, Italy; 5 Scuola Superiore Sant’Anna, Pisa, Italy; 6 Divisione di Radioterapia, Istituto Oncologico Veneto - IRCCS, Padova, Italy; 7 Dipartimento di Scienze Chirurgiche, Oncologiche e Gastroenterologiche (DiSCOG), Università di Padova, Padova, Italy; 8 AP-HP, Hôpital de la Pitié-Salpêtrière, Service de Neurologie 2, Paris, France; 9 Sorbonne Université, INSERM Unité 1127, CNRS UMR 7225, Paris Brain Institute, Equipe labellissée LNCC, Paris, France

**Keywords:** CNS cancer, Prognostic markers, Cancer therapeutic resistance, Apoptosis, Targeted therapies

## Abstract

Glioblastoma (GBM) is an incurable tumor where temozolomide (TMZ) resistance limits survival, even in *MGMT*-methylated patients. To improve stratification, we used NAD(P)H-FLIM to profile TMZ response in 35 patient-derived explants, integrating these data with transcriptomic and functional analyses. We identified *BIRC3* and *CAV1* upregulation in resistant tumors and investigated their parallel yet functionally cooperative role in driving an aggressive, therapy-resistant phenotype. In silico survival analyses demonstrated that *BIRC3* and *CAV1* act as independent prognostic factors whose additive, non-linear effects robustly stratify patient survival beyond *MGMT* status, defining a subgroup with <7% 24-month survival. Importantly, *BIRC3*/cIAP2-driven resistance proved targetable; the IAP antagonist AZD5582 restored TMZ sensitivity by unlocking the apoptotic execution phase, thereby inducing cell death in resistant GBM models in vitro and ex vivo. Our findings establish the *BIRC3/CAV1* axis as a key prognostic signature and therapeutic vulnerability in GBM, offering a new path for precision oncology strategies.

## Introduction

Glioblastoma (GBM) remains incurable despite multimodal standard-of-care, with a median survival of only 15 months [1]. While molecular profiling has refined classification [2], the sole validated biomarker, *MGMT* promoter methylation, is insufficient to predict resistance [3], likely due to extensive intratumoral heterogeneity [4], highlighting the urgent need for novel biomarkers and targets beyond the current standard.

In a previous study [5], we introduced nicotinamide adenine dinucleotide (phosphate) (NAD(P)H) FLIM as a precision medicine tool to evaluate TMZ response in ex vivo GBM explants (GB-EXPs, *n* = 15). This approach allowed us to classify tumors as responder (Resp) or non-responder (Non-Resp) and identify distinct differentially expressed genes associated with resistance. Among these, we identified *BIRC3* as significantly upregulated in TMZ-resistant tumors [5]. *BIRC3* encodes cellular inhibitor of apoptosis 2 (cIAP2), a key apoptosis inhibitor that interferes with caspase activation [6, 7]. Recent studies have implicated BIRC3 in TMZ resistance, showing that its upregulation confers apoptotic evasion in GBM cells and xenografts following TMZ and radiotherapy treatment [8]. Beyond *BIRC3*, our previous analysis also identified *CAV1*, which was significantly upregulated in TMZ-resistant tumors [5]. *CAV1* encodes caveolin-1, a key structural component of caveolae, specialized membrane domains involved in multiple signaling pathways, including cell survival, apoptosis, and drug resistance [9]. While CAV1 has been implicated in tumor progression across various cancer types, its specific role in drug resistance in GBM remains largely unexplored [10]. Several studies suggest that *CAV1* may modulate cellular adhesion, migration, and stress responses, potentially contributing to apoptosis evasion and therapeutic resistance [11, 12].

In this study, we expanded our analysis by employing NAD(P)H-FLIM to assess TMZ response in a larger cohort of primary (*n* = 23) and recurrent (*n* = 12) GB-EXPs. Transcriptomic profiling again confirmed *BIRC3* and *CAV1* upregulation in TMZ-resistant tumors. Building on these findings and addressing these knowledge gaps, this study aimed to: (1) Validate the prognostic significance of *BIRC3* and *CAV1* expression, individually and combined, in large patient cohorts, integrating their value with *MGMT* status. (2) Functionally investigate the cooperative role of *BIRC3* and *CAV1* in driving GBM aggressiveness and TMZ resistance using in vitro models. (3) Explore the potential therapeutic vulnerability of *BIRC3*-driven resistance by testing pharmacological inhibitors of the IAP pathway in vitro and ex vivo.

## Results

### *BIRC3* and *CAV1* are upregulated in TMZ-resistant GBM explants

We analyzed an expanded cohort of 35 patient-derived GBM explants from patients treated according to the Stupp protocol (23 primary, 12 recurrent; Table 1), combining 15 samples from our previous study [5] with 20 newly collected tumors. All samples were assessed for TMZ response using NAD(P)H-FLIM. Using our established NAD(P)H-FLIM protocol [5], samples were stratified into Responders (Resp) and Non-Responders (Non-Resp) based on metabolic shifts following 72 h TMZ treatment.

**Table 1:** Clinical characteristics and TMZ response stratification of the patient-derived GBM cohort.

GB code	Sex	Age	Primary/ recurrence	NADH FLIM TMZ response	Categories DR (FLIM)	IDH 1/2 molecular status	MGMT methylation
GB 1	m	59	P	Resp	MR	WT	unmeth
GB 2	m	57	P	Resp	MR	WT	meth
GB 3	m	30	P	Resp	MR	WT	unmeth
GB 4	f	46	P	Resp	MR	WT	meth
GB 5	m	71	P1	Resp	LR	WT	meth
GB 6	m	65	P2	Resp	HR	WT	meth
GB 7	m	80	P	Resp	HR	WT	meth
GB 8	f	59	P	Non-Resp	NR	WT	unmeth
GB 9	f	59	P	Non-Resp	NR	WT	unmeth
GB 10	f	59	P	Non-Resp	NR	WT	unmeth
GB 11	m	72	P	Non-Resp	NR	WT	unmeth
GB 12	f	40	P	Non-Resp	NR	WT	meth
GB 13	f	73	P	Non-Resp	NR	WT	unmeth
GB 14	m	47	P	Non-Resp	NR	WT	unmeth
GB 15	f	68	P	Resp	LR	WT	meth
GB 16	f	59	R	Resp	LR	WT	unmeth
GB 17	f	68	P	Non-Resp	NR	WT	meth
GB 18	m	72	P	Non-Resp	NR	WT	unmeth
GB 19	m	72	P	Resp	HR	WT	meth
GB 20	m	55	P	Resp	HR	WT	meth
GB 21	f	74	P3	Non-Resp	NR	WT	meth
GB 22	m	76	P	Non-Resp	NR	WT	meth
GB 23	m	73	R1	Resp	HR	WT	meth
GB 24	m	73	R	Resp	HR	WT	nd
GB 25	f	75	R3	Resp	NR	WT	nd
GB 26	m	73	R	Resp	LR	WT	meth
GB 27	m	73	R	Resp	LR	WT	meth
GB 28	f	69	R	Non-Resp	NR	WT	meth
GB 29	f	57	R	Non-Resp	NR	WT	unmeth
GB 30	m	56	R2	Non-Resp	NR	WT	unmeth
GB 31	m	70	R	Non-Resp	NR	WT	unmeth
GB 32	m	70	R	Non-Resp	NR	WT	meth
GB 33	m	57	R	Non-Resp	NR	WT	unmeth
GB 34	m	75	P	Non-Resp	NR	nd	nd
GB 35	f	79	P	Non-Resp	NR	nd	nd

*P* Primary, *R* Recurrence; numbers (e.g., P1/R1) denote matched samples obtained from the same patient at different time points, *WT* Wild-Type, *meth* methylated, *unmeth* unmethylated, *nd* not determined. Categories DR (FLIM): *NR* Non-Responder, *LR* Low Responder, *MR* Medium Responder, *HR* High Responder. Analysis Groups: *Resp* Responder (includes all LR, MR, HR samples), *Non-Resp* Non-Responder (includes NR samples).

Gene expression analysis was performed on 33 samples from the expanded cohort (16 TMZ Resp and 17 TMZ Non-Resp; Table 1), as two samples (GB34, GB35) were excluded due to unavailable RNA for transcriptomic profiling. This analysis revealed that while several genes retained a consistent direction of expression compared to our initial findings, many did not retain statistical significance (Supplementary Data 1). However, we identified a total of 52 genes that remained differentially expressed with statistical significance (adjusted *P* < 0.05) in this larger dataset (Supplementary Data 1).

Among the top ten most statistically significant genes, we confirmed the upregulation of *BIRC3*, which exhibited a 5-fold increase in expression in the TMZ Non-Resp group compared to the Resp group (*P* *adj* = 0.0218, Supplementary Data 1). *CAV1*, which was also identified as a key candidate in our previous work, showed a strong trend of upregulation (a 3-fold increase) in the same global analysis, although it narrowly missed the threshold for statistical significance after correction for multiple comparisons (*P* *adj* = 0.0709, Supplementary Data 1).

**Fig. 1:**
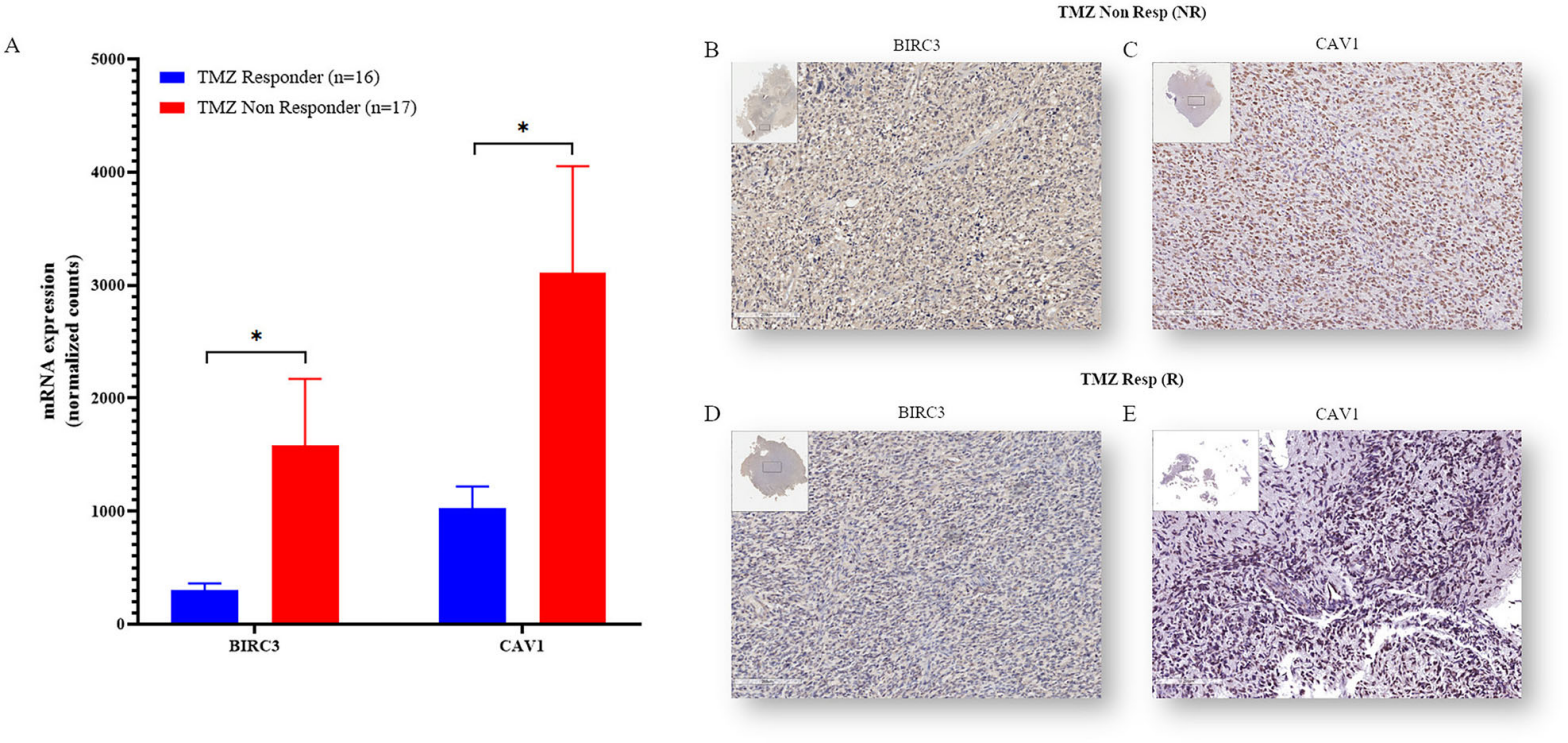
Upregulation of BIRC3 and CAV1 in temozolomide-resistant glioblastoma. **A** Bar plots (mean ± SD) showing *BIRC3* and *CAV1* mRNA expression in sensitive (Resp, *n* = 16) and resistant (Non-Resp, *n* = 17) GBM explants (*P ≤ 0.05). **B**, **C** Representative IHC images of a Non-Responder tumor (strong positivity). **D**, **E** Representative IHC images of a Responder tumor (weak positivity). Scale = 200 µm.

A focused post-hoc comparison using Student’s *t* test confirmed significant upregulation of both *BIRC3* (*P* = 0.0152) and *CAV1* (*P* = 0.0135) in Non-Resp tumors (Fig. 1A), validating their selection for further functional analysis. Upon subdividing the 33-sample transcriptomic cohort into primary (*n* = 21) and recurrent tumors (*n* = 12), we compared *BIRC3* and *CAV1* expression between Resp and Non-Resp. Among primary tumors, there were 10 Resp and 11 Non-Resp; among recurrent tumors, there were 6 Resp and 6 Non-Resp. We conducted a focused post‑hoc analysis comparing *BIRC3* and *CAV1* expression between Resp and Non‑Resp in both primary and recurrent tumor subgroups. As shown in Supplementary Fig. S1A (bar plots of mRNA levels), Non-Resp exhibited consistently higher expression levels of both genes compared to Resp, in both primary and recurrent settings. Although the differences did not achieve statistical significance in this limited dataset (Primary: BIRC3 *P* = 0.067, CAV1 *P* = 0.082; Recurrent: *BIRC3* *P* = 0.176, CAV1 *P* = 0.280), the uniform directionality across subgroups reinforces the potential involvement of *BIRC3* and *CAV1* in modulating TMZ resistance.

Analysis of three matched primary/recurrent pairs (Supplementary Fig. S1B) revealed distinct evolutionary dynamics. Consistent with our hypothesis, acquired resistance (Pair 2: Resp to Non-Resp) coincided with marked *BIRC3*/*CAV1* upregulation, while acquired sensitivity (Pair 3: Non-Resp to Resp) correlated with their downregulation. Although limited by the small sample size, these longitudinal observations provide preliminary evidence linking *BIRC3/CAV1* modulation to the evolution of TMZ sensitivity, generating a hypothesis that warrants validation in larger cohorts.

Consistent with the transcriptomic data, immunohistochemical (IHC) analysis confirmed higher protein expression of cIAP2/BIRC3 and CAV1 in TMZ Non-Responder tumors (Fig. 1B, C) relative to their Responder counterparts (Fig. 1D, E and Supplementary Fig. S2). On average, the percentage of positive cells was significantly higher (assessed by Student’s *t* test) for BIRC3 (~77% vs ~3%, *P* < 0.0001) and CAV1 (~69% vs ~24%, *P* = 0.0039) in Non-Responders compared to Responders (Supplementary Fig. S2).

### *BIRC3* and *CAV1* are highly expressed in GBM and associated with aggressive tumor features

**Fig. 2:**
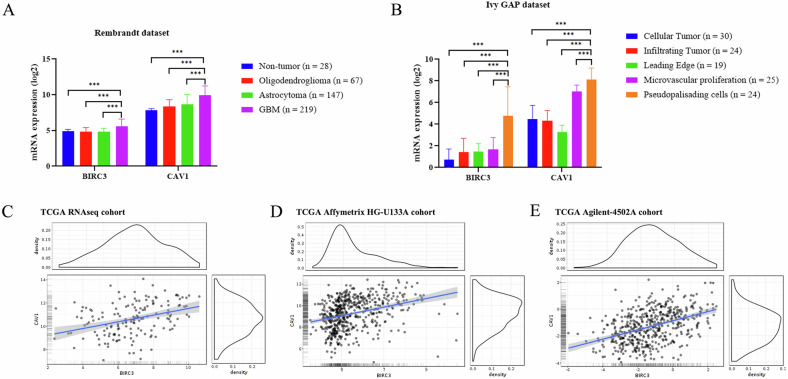
*BIRC3* and *CAV1* expression landscape in Glioblastoma. **A** Expression analysis in glioma subtypes (Rembrandt dataset). **B** Intratumoral localization (IVY-GAP dataset) showing enrichment in pseudopalisading necrosis (***P ≤ 0.001). Scatter plots showing significant positive correlation between *BIRC3* and *CAV1* across TCGA RNA-seq (**C**), Affymetrix (**D**), and Agilent (**E**) datasets.

To explore publicly available datasets on brain tumor expression, we utilized the GlioVis online application for data visualization and analysis (https://gliovis.bioinfo.cnio.es/) [13]. As shown in Fig. 2A, *BIRC3* and *CAV1* expression was analyzed in several gliomas from the Rembrandt dataset [14].

Our analysis reveals a statistically significant upregulation of *BIRC3* and *CAV1* expression in GBM tumors compared to lower-grade gliomas and normal brain tissue. Additionally, the IVY-GAP dataset (IVY Glioblastoma Atlas Project, http://glioblastoma.alleninstitute.org) shows that *BIRC3* and *CAV1* are significantly enriched in pseudopalisading regions surrounding necrosis, a hallmark of aggressive GBM, as illustrated in Fig. 2B.

### *BIRC3* and *CAV1* expression levels show a significant positive correlation across TCGA cohorts

To assess whether *BIRC3* and *CAV1* expression are co-regulated in GBM, we analyzed publicly available TCGA datasets (RNA-seq, *n* = 156; Affymetrix HG-U133A, *n* = 528; Agilent-4502A, *n* = 489) through the GlioVis platform. Pairwise correlation analyses consistently demonstrated a significant positive association between the two genes across all profiling platforms (Pearson r = 0.32–0.44; Spearman ρ = 0.39–0.44; Kendall τ = 0.26–0.30; all *P* < 0.0001) (Fig. 2C–E). These results indicate that *BIRC3* and *CAV1* are co-expressed in GBM, providing a rationale for investigating their combined prognostic impact.

### High *BIRC3* and *CAV1* expression correlates with poor survival in GBM patients

**Fig. 3:**
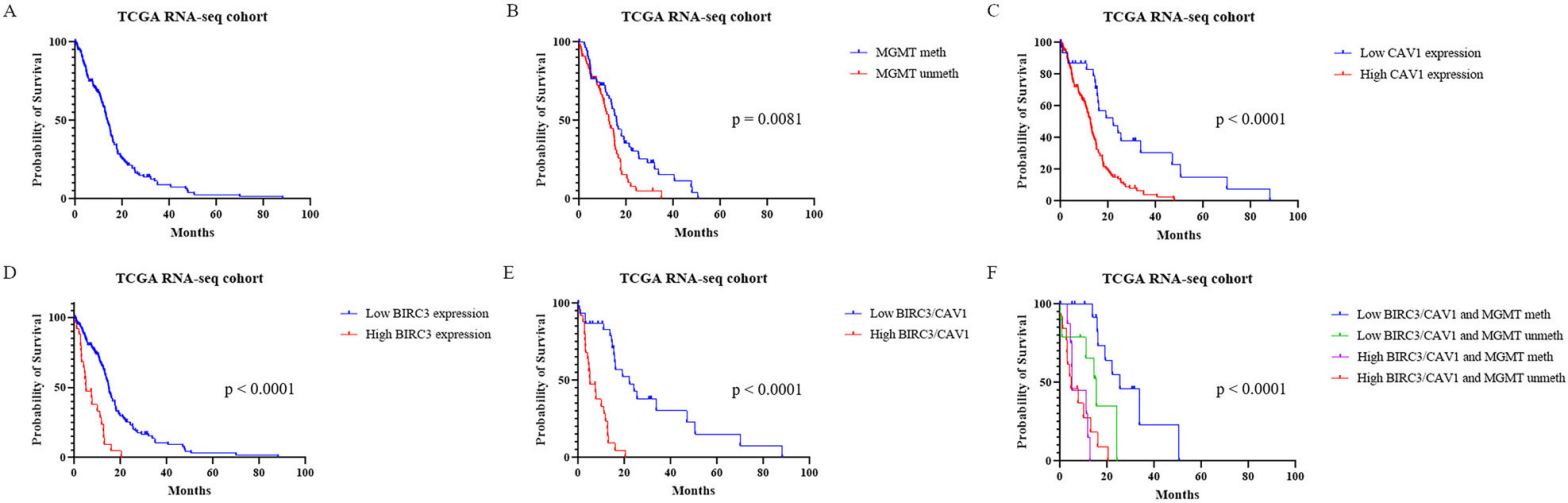
Prognostic value in the TCGA RNA-seq cohort (*n* = 156). Kaplan–Meier curves for Overall Survival (OS). **A** Unstratified cohort. **B** Stratification by *MGMT* methylation. Stratification by *CAV1* (**C**) and *BIRC3* (**D**) alone. **E** Combined *BIRC3*/*CAV1* stratification. **F** Integrated model with *MGMT* status. *P*-values by Log-rank test.

In the TCGA RNA-seq cohort (*n* = 156), the median OS was 13.6 months (Fig. 3A). Stratification by *MGMT* promoter methylation alone confirmed its prognostic value (Fig. 3B). Kaplan–Meier analysis revealed that high expression of *BIRC3* or *CAV1* was significantly associated with shorter OS (Log-rank *P* < 0.0001; Fig. 3C, D). The “High *BIRC3*/High *CAV1*” subgroup exhibited the poorest prognosis (median OS 5.1 months) compared to the “Low/Low” group (22.2 months) (Fig. 3E). Integrating gene expression with *MGMT* status defined four prognostic groups: notably, *BIRC3*/*CAV1* stratification identified a subset of *MGMT*-unmethylated patients with exceptionally poor survival (4.6 months; Fig. 3F).

### Cox regression analyses confirm *BIRC3* and *CAV1* as independent prognostic biomarkers in GBM

To assess whether BIRC3 and CAV1 provide prognostic information independent of MGMT promoter methylation, we performed multivariate Cox regression across three TCGA platforms (Supplementary Fig. S3). In the RNA-seq cohort, both BIRC3 (HR = 1.97, *P* = 0.013) and CAV1 (HR = 2.61, *P* < 0.001) remained significant predictors after adjusting for MGMT status. This independence was validated in the Agilent-4502A cohort (BIRC3: HR = 1.84; CAV1: HR = 1.34) and partially in the Affymetrix HG-U133A cohort, where BIRC3 retained significance (HR = 1.52) while CAV1 did not. Collectively, these data demonstrate that BIRC3 and CAV1 offer prognostic value distinct from MGMT methylation.

### Combined prognostic impact of *BIRC3* and *CAV1* expression reveals additive risk in GBM

Combined prognostic impact and non-linear risk modeling Analysis of the RNA-seq cohort revealed that co-upregulation of *BIRC3* and *CAV1* is associated with a drastically reduced 12-month survival probability (23% vs 83% in the double-low group; Supplementary Table S1). Additive Cox models confirmed that both genes contribute independently to mortality (Combined HR ≈ 7.31 versus double-low), with no statistically significant multiplicative interaction.

To capture complex risk dynamics, we employed restricted cubic splines (Supplementary Fig. S4) and quantified hazards at extreme percentiles (Supplementary Table S2). Comparing the 90th vs 10th percentiles, *BIRC3* alone significantly increased risk (HR = 2.14, *P* = 0.005), whereas *CAV1* alone did not reach significance (HR = 1.45, *P* = 0.113). Crucially, the combined high-expression profile (90th/90th) exerted the strongest effect (HR = 2.67, *P* = 0.0021).

Supplementary Table S3 illustrates the clinical translation of this model: while patients with an optimal profile (*BIRC3* 10th/*CAV1* 50th) had a 24-month survival (S_24_) of 44%, those in the high-risk group (*BIRC3* 90th/*CAV1* 90th) dropped to an S_24_ of only 6.9%. Prognostic significance was confirmed across independent datasets and different measurement platforms. To do this, we performed multivariable Cox regression analyses adjusted for *MGMT* promoter methylation status, with the results summarized in forest plots (Supplementary Fig. S5).

As shown in Supplementary Fig. S5, high BIRC3 expression was associated with increased mortality in the discovery cohort (TCGA RNA-seq, *n* = 156; random-effects pooled HR = 1.26 [1.06–1.50]), and this association was successfully replicated in the validation cohort (TCGA Agilent-4502A, *n* = 489; pooled HR = 1.30 [1.11–1.52]).

A similar consistent effect was observed for high *CAV1* expression. The combined linear predictor (LP) integrating both markers also retained strong prognostic value across all analyses. Importantly, the direction and magnitude of these effects were consistent across independent cohorts and transcriptomic platforms, supporting the robustness of the *BIRC3*/*CAV1* prognostic signature.

### The prognostic value of *BIRC3* and *CAV1* is validated in independent European cohorts

To validate the prognostic value of *BIRC3* and *CAV1* expression observed in public datasets, we analyzed two independent European cohorts of *IDH*-wildtype GBM patients treated according to the Stupp protocol: a French cohort (*n* = 228) analyzed by RNA sequencing (Supplementary Data 2) and an Italian cohort (*n* = 68) analyzed by RT-qPCR (Supplementary Data 3). The French cohort included only primary tumors, while the Italian cohort consisted of 48 primary and 20 recurrent cases.

**Fig. 4:**
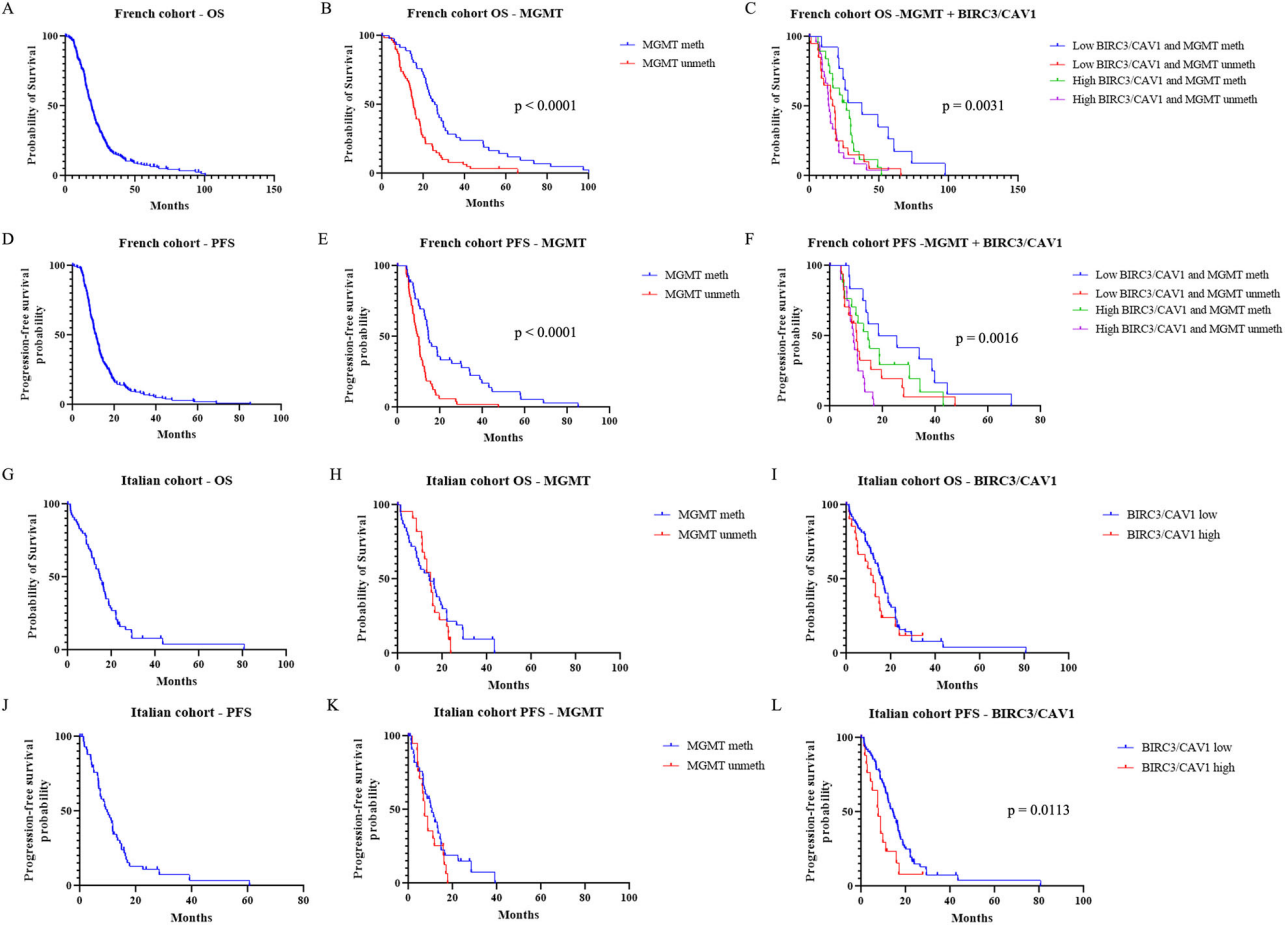
Validation in independent European cohorts. **A**–**F** Analysis of the French Cohort (*n* = 228, RNA-seq). Kaplan–Meier curves for Overall Survival (**A**) and Progression-Free Survival (**D**). Stratification by *MGMT* promoter methylation status alone for OS (**B**) and PFS (**E**). Combined stratification by *MGMT* status and *BIRC3*/*CAV1* expression defining four prognostic groups for OS (**C**) and PFS (**F**). **G**–**L** Analysis of the Italian Cohort (*n* = 68, RT-qPCR). Kaplan–Meier curves for Overall Survival (**G**) and Progression-Free Survival (**J**). Stratification by *MGMT* methylation status for OS (**H**) and PFS (**K**). Stratification by the *BIRC3*/*CAV1* signature alone (high vs low) for OS (**I**) and PFS (**L**), showing a statistically significant impact on progression-free survival (*P* = 0.0113). *P*-values determined by Log-rank (Mantel–Cox) test.

In the French cohort, the median overall survival (OS) and progression-free survival (PFS) were 18.57 and 11.2 months, respectively (Fig. 4A, D). Stratification by *MGMT* promoter methylation status alone confirmed its prognostic relevance, with methylated tumors displaying significantly improved outcomes (OS: 25.5 vs 15.13 months, PFS: 14.57 vs 9.37 months; *P* < 0.0001 for both; Fig. 4B, E).

However, incorporating *BIRC3* and *CAV1* expression, dichotomized using median values (5.9 for *BIRC3* and 7.5 for *CAV1*), provided additional prognostic resolution. Patients with low expression of both genes had significantly better survival compared to those with high expression (OS: 18.8 vs 15.97 months; PFS: 12.6 vs 9.57 months; data not shown). More importantly, combining *BIRC3*/*CAV1* expression with *MGMT* methylation status revealed four distinct prognostic subgroups (Fig. 4C–F). The most favorable outcome was observed in patients with low *BIRC3*/*CAV1* expression and methylated *MGMT* (OS: 37.83 months; PFS: 22 months), while those with high gene expression and unmethylated *MGMT* had the worst prognosis (OS: 13.97 months; PFS: 8.88 months). This stratification was statistically significant for both OS and PFS (*P* < 0.01). These findings highlight the added value of assessing *BIRC3* and *CAV1* expression in conjunction with *MGMT* status to better define long- and short-term survivors among GBM patients.

In the smaller Italian GBM cohort, the median overall survival (OS) and progression-free survival (PFS) were 14.67 and 9.73 months, respectively (Fig. 4G–I). Stratification based on *MGMT* promoter methylation status did not result in statistically significant differences in survival, although a trend toward better outcome was observed in the methylated group (OS: 14.3 vs 14.64 months; PFS: 10.6 vs 7.27 months; *P* = ns; Fig. 4H–K).

Crucially, when patients were stratified using the *BIRC3*/*CAV1* signature, we found a strong and statistically significant impact on Progression-Free Survival (PFS), a key clinical endpoint that measures time to tumor progression. The low-expression group exhibited a median PFS nearly double that of the high-expression group (14.3 vs 7.6 months; *P* = 0.0113; Fig. 4L).

A clear trend in the same direction was observed for Overall Survival (15.99 vs 12.1 months), which approached but did not reach statistical significance (*P* = 0.0990; Fig. 4I), likely reflecting the limited statistical power of this smaller cohort. Nevertheless, the robust association with PFS provides strong, independent validation from a clinically relevant perspective, reinforcing the role of the *BIRC3*/*CAV1* signature in predicting tumor aggressiveness and treatment outcome.

### Co-expression of *BIRC3* and *CAV1* drives an aggressive phenotype

**Fig. 5:**
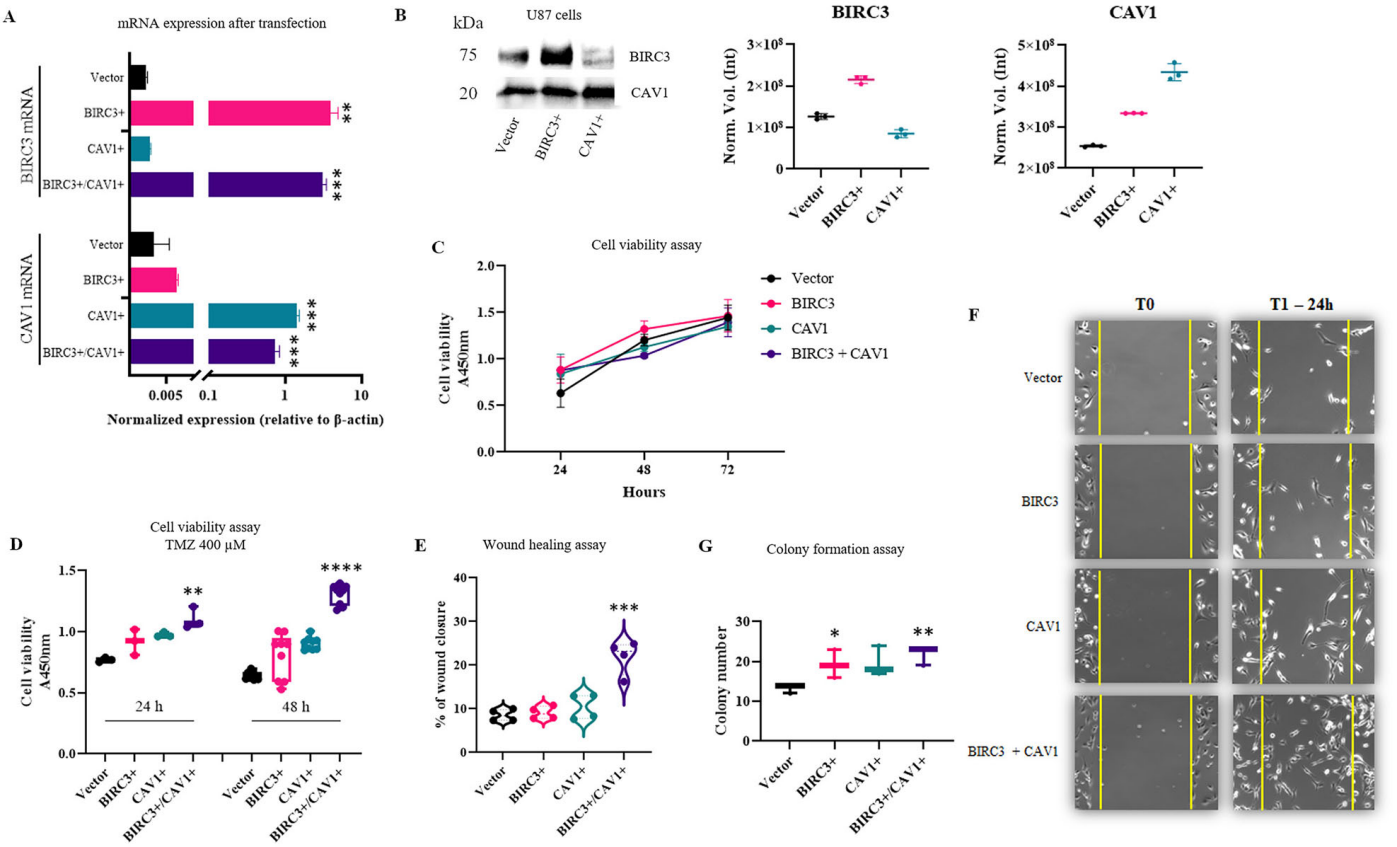
Molecular characterization and functional impact of *BIRC3* and *CAV1* overexpression in TMZ-sensitive U87 cells. **A** RT-qPCR validation of *BIRC3* and *CAV1* overexpression showing transfection efficiency (** *P* < 0.01; ***P < 0.001 vs Vector). **B** Representative Western blot analysis and densitometric quantification of cIAP2/BIRC3 and CAV1 protein levels in single-transfected cells. Protein volumes were normalized to total protein content using Stain-Free imaging technology. **C** Basal cell viability assessed by WST-1 assay over 72 h. **D** Cell viability after 400 µM TMZ treatment (24–48 h) (**P < 0.01; ****P < 0.0001 vs Vector). **E** Quantification of migration potential by wound healing assay (***P < 0.001). **F** Representative images of wound closure at 0 and 24 h. **G** Colony formation assay quantification (*P < 0.05; **P < 0.01). Data represent mean ± SD of three independent biological replicates.

To establish causality, we performed gain-of-function studies in TMZ-sensitive U87 cells [3]. Transient transfection with *BIRC3*, *CAV1*, or both confirmed significant upregulation of their respective transcripts (Fig. 5A, *P* < 0.01) and protein levels (Fig. 5B and Supplementary Table S4, *P* < 0.001).

Importantly, Western blot analysis revealed that while ectopic expression of each gene robustly increased its respective protein levels, the cross-modulation between the two proteins was minimal compared to the magnitude of their direct overexpression. This confirms that BIRC3 and CAV1 do not operate in a strict upstream/downstream hierarchical manner, but instead act predominantly through parallel pathways to synergistically drive the aggressive phenotype.

We next evaluated the functional impact of this overexpression. Under basal conditions, cell viability was unaffected (Fig. 5C). However, following TMZ treatment (400 µM), *BIRC3*/*CAV1* co-expression significantly protected cells from cytotoxicity compared to vector control (*P* < 0.0001 at 48 h; Fig. 5D), suggesting a cooperative resistance mechanism. Consistent with this aggressive profile, wound healing assays revealed that *BIRC3/CAV1* cells exhibited a significantly higher migration rate than all other conditions (*P* = 0.0007; Fig. 5E, F), indicating an aggressive phenotype. Co-expression significantly enhanced long-term clonogenic potential (*P* = 0.0050; Fig. 5G).

To investigate the level of blockade, we analyzed the transcriptional response of apoptotic markers in U87 overexpressing cells (Supplementary Fig. S6). Following TMZ treatment, *BAX* mRNA showed a consistent upward trend across all conditions, reaching statistical significance in Vector and *BIRC3+* cells (*P* < 0.05). Conversely, the anti-apoptotic marker *BCL2* exhibited a downregulation trend in all treated groups, which was statistically significant in *BIRC3*-overexpressing cells (*P* < 0.05). Regarding *CASP3*, we observed a divergent pattern: while TMZ treatment significantly reduced *CASP3* mRNA levels in sensitive Vector control cells (*P* < 0.05), it induced an increasing trend in *BIRC3+* and *BIRC3*+*/CAV1+* resistant cells. The observation that resistant cells maintain high levels of pro-apoptotic transcripts (upregulated *BAX*, sustained *CASP3*) and reduced *BCL2* confirms that the upstream damage sensing and transcriptional priming are intact. This apparent paradox suggests that the *BIRC3*/*CAV1* axis overrides this pro-death signal downstream, prompting us to investigate the execution phase at the protein level.

### The Smac-mimetic AZD5582 restores TMZ sensitivity in resistant cells by unblocking caspase activity

To pharmacologically reverse the identified resistance, we targeted BIRC3 using AZD5582, a potent pan-IAP antagonist described by Hennessy et al. [15]. Since IAP inhibition creates a dependency that sensitizes tumors to cytotoxic agents, we evaluated AZD5582 both as monotherapy and in combination with TMZ [16, 17].

**Fig. 6:**
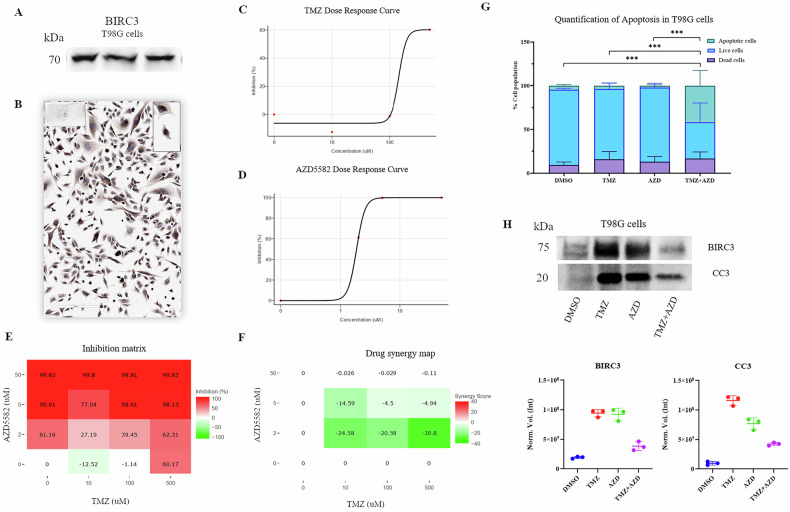
Reversal of TMZ resistance by AZD5582 in TMZ-resistant T98G cells. Basal expression of cIAP2/BIRC3 in T98G cells evaluated by Western blot (**A**) and immunocytochemistry (**B**); the red box indicates the specific band at ~70 kDa. **C**, **D** Dose-response curves for TMZ and AZD5582 after 72 h treatment. **E** Inhibition matrix showing the percentage of cell growth inhibition for TMZ and AZD5582 combinations relative to DMSO control. **F** Drug synergy map calculated using the ZIP model; green regions indicate antagonism (score < −10). **G** Flow cytometry analysis of Annexin V-FITC/PI staining at 72 h; cell populations are color-coded as Live (blue), Early Apoptotic (teal), and Dead (purple). **H** Representative Western blot and densitometric quantification of cIAP2/BIRC3 and Cleaved Caspase-3 (CC3) protein levels; volumes were normalized to total protein load via Stain-Free imaging. Data represent mean ± SD of three independent biological replicates (***P ≤ 0.001).

We utilized T98G cells, characterized by intrinsic TMZ resistance [3]. Western blot and immunocytochemistry confirmed high basal expression of cIAP2 (BIRC3) in both cytoplasmic and nuclear compartments (Fig. 6A, B). While AZD5582 displayed potent single-agent growth inhibitory activity (Fig. 6D), proliferation assays suggested an antagonistic interaction with TMZ (Synergy scores < −10; Fig. 6F). Direct apoptosis assessment resolved this apparent paradox: while TMZ (500 µM) AZD5582 (5 µM) alone failed to induce significant cell death, the combination (TMZ 500 µM + AZD5582 5 µM) significantly increased early apoptotic events (Annexin V+/PI− cells, ~40%) compared to single agents (*P* < 0.001; Fig. 6G). To mechanistically investigate the execution phase, we analyzed Caspase-3 at the protein level (Fig. 6H).

Western blot analysis revealed that TMZ treatment alone induced a marked accumulation of Cleaved Caspase-3, coinciding with the strong upregulation of BIRC3.

In contrast, the combination with AZD5582 caused the depletion of BIRC3 and resulted in lower steady-state levels of Cleaved Caspase-3 compared to TMZ alone, while significantly inducing apoptosis (Annexin V+ cells), consistent with dynamic caspase activation during apoptotic execution. Densitometric quantification (Supplementary Table S5) confirmed that the combination of TMZ and AZD5582 leads to a drastic reduction in BIRC3 protein levels (~60% decrease compared to TMZ monotherapy), while maintaining Cleaved Caspase-3 at levels significantly higher than DMSO control.

### *BIRC3* levels predict the therapeutic efficacy of IAP inhibition in patient-derived tumor explants

**Fig. 7:**
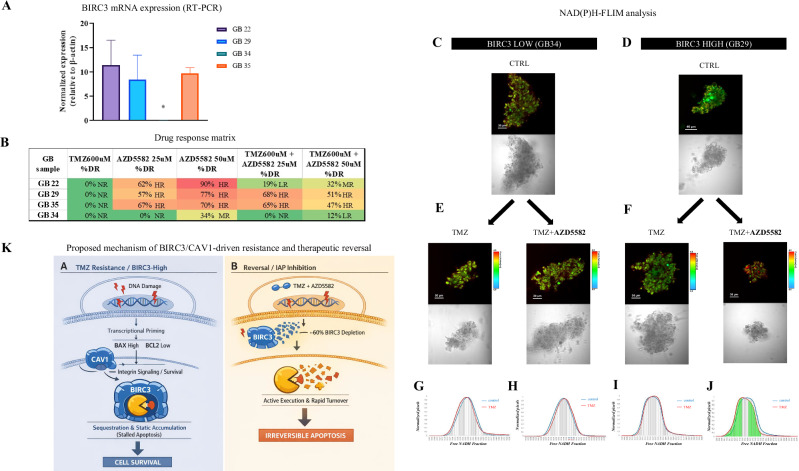
Ex vivo validation of therapeutic targeting and proposed mechanism. **A** *BIRC3* mRNA levels in four patient tumors. **B** Drug response (%DR) measured by NAD(P)H-FLIM. **C**–**J** Representative FLIM maps and brightfield images. Note the significant metabolic shift in the *BIRC3*-high tumor (GB29, panel **J**) following combination treatment, absent in the *BIRC3*-low tumor (GB34, panel **H**). **K** Proposed mechanism of BIRC3/CAV1-driven resistance and therapeutic reversal. TMZ Resistance (left): In resistant GBM cells, BIRC3 prevents cell death by sequestering cleaved Caspase-3 in stable complexes, leading to a static accumulation of the protease and a state of stalled apoptosis. CAV1 operates in a predominantly parallel pathway to promote tumor aggressiveness. Therapeutic Reversal (right): AZD5582 triggers a ~60% depletion of BIRC3, effectively removing the molecular brake on the execution phase. Upon BIRC3 degradation, cleaved Caspase-3 is released to execute cell death. The subsequent reduction in steady-state Caspase-3 protein levels reflects its active enzymatic consumption and rapid turnover during irreversible apoptosis.

We validated this strategy ex vivo on four tumors characterized by FLIM [5] (Fig. 7A). In *BIRC3*-high tumors (GB22, GB29, GB35), AZD5582 (25–50 µM) successfully restored TMZ sensitivity, converting Non-Responders to Responders (Fig. 7B). Conversely, the *BIRC3*-low tumor (GB34) showed minimal benefit. Metabolic imaging (Fig. 7C–J) confirmed a drug-induced shift only in *BIRC*3-high tumors, positioning *BIRC3* as a predictive biomarker for IAP-inhibitor efficacy.

Collectively, our findings are summarized in the proposed mechanism of resistance and therapeutic reversal depicted in Fig. 7K. In resistant GBM cells (Fig. 7K, left), TMZ induces a pro-apoptotic transcriptional program (High *BAX*, Low *BCL2*), but cell death is prevented by high levels of *BIRC3*, which sequesters cleaved Caspase-3 in stable, non-functional complexes. This leads to a static accumulation of the processed protease without inducing apoptosis, a state defined as stalled apoptosis. In this context, *CAV1* operates in a predominantly parallel pathway to promote an aggressive and migratory phenotype. Conversely, during therapeutic reversal (Fig. 7K, right), treatment with the Smac-mimetic AZD5582 degrades BIRC3, removing the molecular brake on the execution phase. Upon BIRC3 depletion, cleaved Caspase-3 is released to actively execute cell death. The observed reduction in Caspase-3 protein levels reflects the enzymatic consumption and rapid turnover of the enzyme during irreversible apoptosis.

## Discussion

Glioblastoma (GBM) remains a clinical challenge due to its extensive heterogeneity [4, 18] and intrinsic resistance to apoptosis. While *MGMT* promoter methylation is the standard predictive biomarker, its utility is limited by discordant cases and lack of standardized cutoffs [19–21]. In this study, we employed a “function-first” discovery strategy using NAD(P)H-FLIM imaging [5, 22] to identify a molecular signature derived directly from the metabolic response of live patient tumors. We report that the cooperative upregulation of *BIRC3* and *CAV1* drives a therapy-resistant phenotype and defines a patient subgroup with dismal prognosis, offering a robust alternative to standard stratification.

Our transcriptomic analysis identified BIRC3 (cIAP2) as the most significant IAP family member associated with resistance. While BIRC3 is a known inhibitor of apoptosis [6–8] and CAV1 is linked to invasion [10, 12], their combined impact in GBM was previously unexplored. By integrating these markers, we achieved a stratification power significantly superior to MGMT status alone. Multivariate Cox analysis across three independent TCGA cohorts confirmed that both genes are independent prognostic factors. Notably, our use of restricted cubic splines revealed a previously unappreciated complexity in CAV1 signaling: we observed a U-shaped risk profile in multivariable spline modeling, where both deficiency and overexpression are detrimental. This non-linearity likely explains why previous studies on CAV1 yielded inconsistent results [23], underscoring the need for advanced modeling. The clinical implication is profound: patients with the “double-high” signature face a <7% probability of survival at 24 months, identifying an ultra-high-risk population.

Mechanistically, our gain-of-function studies in U87 cells provide the causal link between this signature and the resistant phenotype. We demonstrated that BIRC3/CAV1 co-expression shields typically sensitive cells from chemotherapy-induced death. Transcriptional analysis of these overexpressing cells revealed that TMZ treatment correctly triggered a comprehensive pro-apoptotic program, evidenced by the significant upregulation of the effector BAX and the downregulation of anti-apoptotic BCL2. However, this potent signaling failed to translate into cell death. This discrepancy suggests that the resistance mechanism driven by the BIRC3/CAV1 axis operates by uncoupling upstream damage sensing from the final execution phase. A key question addressed in our study was the hierarchical relationship between these two markers. Our cross-modulation experiments demonstrated that ectopic expression of BIRC3 does not regulate CAV1 levels and vice versa, supporting a model of parallel pathway activation. While we cannot exclude that CAV1 may influence the localization of upstream death receptors, its primary role in our model appears independent of BIRC3’s direct inhibitory action on executioner caspases. This parallel architecture is particularly relevant for therapeutic targeting: by acting on distinct nodes of the resistance network, with BIRC3 blocking the execution phase [24, 25] and CAV1 potentially modulating integrin-mediated survival and migration [12], this axis provides multiple layers of protection against therapy-induced stress. This explains why their co-expression defines a subgroup of patients with such exceptionally poor prognosis.

To functionally validate this independent blockade mechanism and explore its therapeutic targeting, we utilized the TMZ-resistant T98G model, which exhibits high basal levels of BIRC3, acting as a post-translational brake on executioner caspases [24]. Investigating the execution phase at the protein level revealed distinct molecular dynamics: T98G cells treated with TMZ alone exhibited a paradoxical accumulation of processed Cleaved Caspase-3 yet failed to undergo apoptosis. This accumulation is consistent with the established role of cIAP2/BIRC3 as an E3 ubiquitin-ligase capable of sequestering the processed caspase in stable, non-functional complexes without inducing its immediate degradation [24]. Consequently, the apoptotic machinery is primed but functionally blocked at the execution step, a phenomenon described as stalled apoptosis [25].

Pharmacological targeting of this mechanism using the pan-IAP antagonist AZD5582 [15] confirmed this hypothesis. In T98G GBM cells, the persistence of BIRC3 protein levels following monotherapy may be explained by a compensatory feedback loop; in cancers, activation of NF-κB increases the expression of these IAPs to maintain cell survival [26]. Specifically, Smac-mimetic induced degradation of cIAP1 triggers non-canonical NF-κB signaling, which results in the de novo synthesis of cIAP2 [27].

However, the synergistic stress induced by combination with TMZ overrides this compensatory effort. Our protein-level analysis (confirmed by a ~60% reduction in BIRC3) demonstrates that the synergy is mediated by the depletion of the BIRC3 brake. This depletion effectively removes the post-translational blockade, allowing the activated pool of Caspase-3 to be released from sequestration and trigger irreversible apoptosis. This transition is further evidenced by the shift from a static accumulation of the protease to its active enzymatic consumption and rapid proteolytic turnover [28]. Unlike the resistance phase, the transition to sensitivity is characterized by the active consumption of the enzyme during the final stages of cell death, explaining the lower steady-state levels of CC3 observed in the combination group concomitant with massive Annexin V positivity.

A pivotal finding regarding the interaction between TMZ and AZD5582 further clarifies the therapeutic potential. While proliferation assays suggested an antagonistic interaction, direct apoptosis assessment revealed that the combination shifted the cellular fate from simple cytostatic arrest to irreversible cell death. This highlights a critical limitation of conventional synergy models in the context of IAP antagonists: the therapeutic goal in these aggressive tumors is not merely cytostasis, but the induction of apoptosis, which AZD5582 achieves by removing the brake imposed by BIRC3 [29]. Building on these mechanistic findings, we performed ex vivo validation on four patient-derived tumors using FLIM imaging. In *BIRC3*-high tumors, AZD5582 successfully restored TMZ sensitivity, whereas *BIRC3*-low tumors showed minimal response. Although limited by the small sample size (*n* = 4), this exploratory analysis provides proof-of-concept support that *BIRC3*/*CAV1*-high tumors may represent a therapeutically vulnerable subgroup, warranting further investigation in larger patient cohorts. Finally, our exploratory analysis on paired samples suggests a potential enrichment of the BIRC3/CAV1 signature at recurrence, a hypothesis-generating observation requiring validation in larger longitudinal cohorts.

In conclusion, we establish the BIRC3/CAV1 axis as a key prognostic signature and an actionable therapeutic target. By integrating functional imaging with molecular profiling, this study provides a framework for precision oncology, positioning BIRC3 as a predictive beacon for selecting patients who may benefit from IAP-targeted therapies.

## Materials and methods

### Patient cohorts and ethics

The study was conducted in accordance with the Declaration of Helsinki. Samples from the Italian cohort (*n* = 68) were obtained under approval from the University Hospital of Pisa Ethics Committee (787/2015). The French cohort (*n* = 228) was collected at Pitié-Salpêtrière Hospital (Paris) with ethical approval (OncoNeuroTek). All patients provided informed consent. All patients underwent standard radiochemotherapy (Stupp protocol). Clinical data are summarized in Table 1. No specific inclusion/exclusion criteria were applied beyond the diagnosis of GBM and availability of follow-up data.

### Cell culture and patient-derived explants (GB-EXPs)

U87 and T98G cell lines were obtained from ATCC and authenticated by STR profiling within the last three years. All cells were routinely tested for mycoplasma contamination. Cells were cultured in DMEM/10% FBS. Patient-derived tumors were dissociated using the gentleMACS Dissociator (Miltenyi Biotec) and cultured as 3D explants in Matrigel as previously described [5].

### NAD(P)H-FLIM metabolic imaging

FLIM experiments were performed using a MultiHarp 150 TCSPC unit (Picoquant) coupled to a confocal microscope (excitation 405 nm; emission 440/40 nm) as described [5, 30]. Phasor analysis was conducted using SimFCS software (Laboratory for Fluorescence Dynamics). Drug response (%DR) was calculated based on the shift in metabolic trajectory relative to untreated controls [5].

### Gene and protein expression

Total RNA was extracted (Maxwell 16, Promega) and quantified. Libraries were prepared using Illumina Stranded Total RNA Prep (Italian cohort) or QuantSeq 3′ mRNA-Seq (French cohort) and sequenced on Illumina platforms. For RT-qPCR, cDNA was synthesized from 250 ng of total RNA using the iScript™ cDNA Synthesis Kit (Bio-Rad, Hercules, CA, USA). Quantitative PCR was performed with Universal SYBR Green Supermix (Bio-Rad) on a thermal cycler. Ct values for target genes were normalized to the housekeeping gene β-actin. Primers used were as follows: BIRC3 forward GATCCATGGGTTCAA, reverse GGCTTGAACTTGACG; CAV1 forward TCTCTACACCGTTCCCATCC, reverse TGCCGTCAAAACTGTGTGTC; BAX forward TTTGCTTCAGGGTTTCATCC, reverse CAGTTGAAGTTGCCGTCAGA; BCL2 forward GAACTGGGGAGGATTGTGG, reverse GGCCAAACTGAGCAGAGTCT; CASP3 forward GGTTCTGGAGGATTTGGTGA, reverse AGCACCATTTTCTTGGCAGT; and β-actin forward CTGGCACCACACCTTCTAGA, reverse GTACATGGCTGGGGTGTTGA. For protein analysis, IHC was performed on paraffin sections using anti-BIRC3 (Merck, HP002317) and anti-CAV1 (Life Technologies, MA3-600) antibodies. Signals were quantified using QuPath [31]. Western blot analysis was performed on whole-cell lysates. For basal expression profiling, proteins were probed with anti-BIRC3 (Merck, HP002317). For functional validation, U87 cells were lysed 48 h post-transfection, whereas T98G cells were lysed 72 h post-treatment. Proteins were separated on Mini-PROTEAN TGX Stain-Free gels (Bio-Rad) and transferred to PVDF membranes. Total protein load was captured immediately after transfer for stain-free normalization.

To ensure accurate comparison and consistent protein loading across different targets, proteins from the same lysate were transferred onto a single PVDF membrane. Membranes were subsequently horizontally cut to allow for the simultaneous detection of high-molecular-weight targets (BIRC3, ~70 kDa) and low-molecular-weight targets (CAV1 or Cleaved Caspase-3, <37 kDa). Blots were probed with the following rabbit monoclonal antibodies: anti-c-IAP2 (Clone 58C7, Cell Signaling Technology #3130), anti-Caveolin-1 (Clone D46G3, Cell Signaling Technology #3267), and anti-Cleaved Caspase-3 (Asp175, Cell Signaling Technology #9661). Detection was performed using HRP-conjugated Goat Anti-Rabbit IgG (Abcam ab7090) and developed with Clarity Western ECL Substrate (Bio-Rad). To ensure maximum accuracy, BIRC3, CAV1, and CC3 protein levels were assessed using Stain-Free densitometric quantification; detailed data are reported in Supplementary Table S5. All Western blot experiments were performed in three independent biological replicates to ensure reproducibility.

### Functional assays and transfection

U87 cells were transiently transfected with GFP-tagged ORF plasmids (Origene: BIRC3 RG211599; CAV1 RG210274) using TurboFectin 8.0.

- *Viability:* Assessed via RealTime-Glo (Promega) or WST-1 (Merck) at 24–72 h.
- *Clonogenicity:* Colonies (>50 cells) were counted after 14 days.
- *Migration:* Assessed by wound healing assay at 24 h.
- *Apoptosis:* T98G cells were stained with Annexin V-FITC/PI (Beckman Coulter) after 72 h treatment with AZD5582 (5 µM; Sigma-Aldrich, SML2900) alone or in combination with TMZ (500 µM; Sigma-Aldrich, T2577) and analyzed by flow cytometry. Drug combination effects were analyzed using the SynergyFinder 3.0 web application [32]. Synergy scores were calculated using the ZIP (Zero Interaction Potency) reference model. A synergy score >10 indicates synergy, between −10 and 10 indicates additivity, and <−10 indicates antagonism. All in vitro functional assays were performed in at least three independent biological replicates.

### Statistical and bioinformatic analysis

RNA-seq data were analyzed using DESeq2 [33]. Public datasets (TCGA, Rembrandt, IVY-GAP) were accessed via GlioVis [13]. Survival analysis was performed using R software (v4.2.0) with *survival* and *rms* packages [33]. We used Kaplan–Meier methods (Log-rank test) and multivariate Cox proportional hazards models. Non-linear effects were modeled using restricted cubic splines. Normality was assessed prior to parametric testing, and variance was comparable between groups. Two-tailed Student’s *t* test was used for group comparisons (GraphPad Prism). *P* < 0.05 was considered significant. Data are presented as mean ± Standard Deviation (SD). Sample sizes were determined based on prior experience and availability of patient-derived material; sample sizes were determined based on data availability and standard experimental protocols in the field. Randomization and blinding were not applicable to the experimental design.

## Supplementary information

SUPPLEMENTARY MATERIAL

Supplementary Data 1

Supplementary Data 2

Supplementary Data 3

Original Blots

## Data Availability

The datasets generated and/or analyzed during the current study, including the data for the Italian and French cohorts, are provided in the supplementary tables accompanying this published article. Publicly available datasets were also analyzed in this study. The raw data for the TCGA, IVY-GAP, and Rembrandt cohorts can be found in their respective public repositories.

## Code availability

The custom code and analysis scripts used to generate the results reported in this manuscript are available from the corresponding author on reasonable request. The software packages and R libraries used are detailed in the “Materials and Methods” section and are publicly available.
