# Supplementary material for: BIRC3/CAV1 co-expression drives GBM aggressiveness as a prognostic signature and therapeutic vulnerability
Source: Cell Death Discov. 2026 Apr 14;12:232. doi: 10.1038/s41420-026-03112-z (PMC13183958; doi:10.1038/s41420-026-03112-z)
Supplement: Supplementary file 1 — SUPPLEMENTARY MATERIAL [file 41420_2026_3112_MOESM1_ESM.docx]

**SUPPLEMENTARY MATERIAL**

**Figure S1**. BIRC3 and CAV1 expression dynamics stratified by tumor origin and in matched pairs. **A**, Expression of BIRC3 and CAV1 mRNA stratified by tumor origin (Primitive, n = 21 vs. Recurrent, n = 12) and response to TMZ. Statistical significance was assessed using Student's unpaired t-test. **B**, mRNA expression levels of *BIRC3* (blue) and *CAV1* (red) in three matched pairs of primary (P) and recurrent (R) tumors from the same patients. TMZ response status is indicated for each sample (Resp = Responder; Non-Resp = Non-Responder). These data identify trends in BIRC3/CAV1 modulation during tumor evolution, supporting the hypothesis of their involvement in acquired resistance.


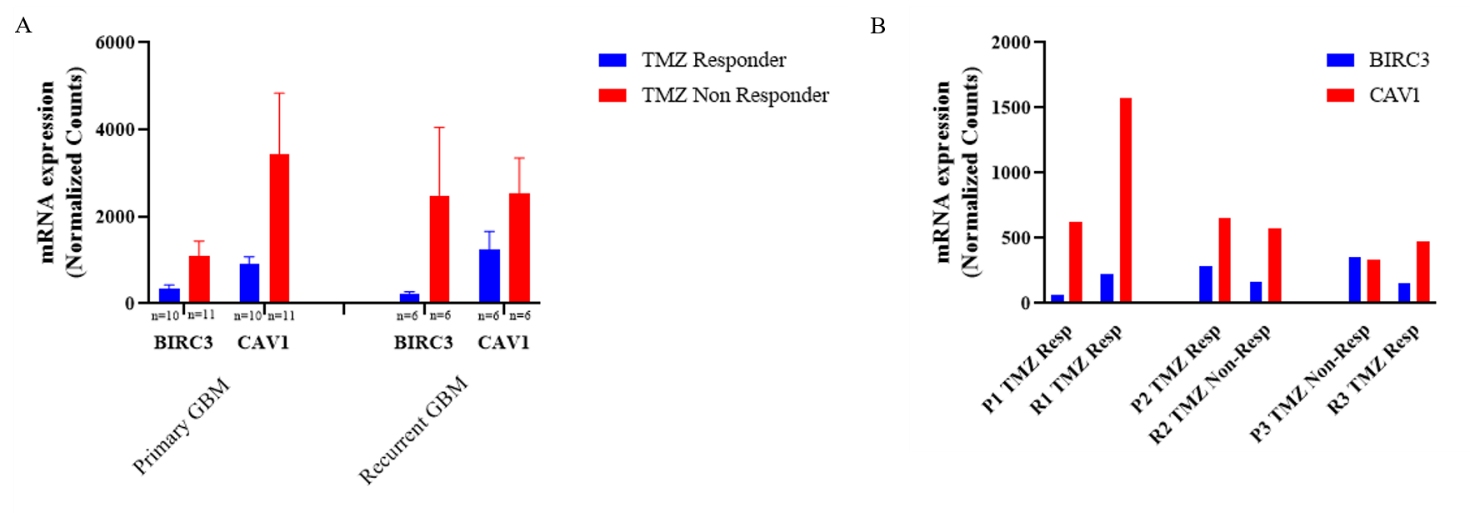


**Figure S2**: Quantification of BIRC3 and CAV1 protein expression in responder and non-responder tumors. Five/ six areas of 200 µm² were chosen within each image of a TMZ responder and Non responder tissues. cIAP2 and CAV1 signals were quantified as % of positive cells with QuPath (<https://qupath.github.io/>). Statistical comparisons were performed using Student's two-tailed unpaired t-test. The statistical results of the comparison are reported in the main text.


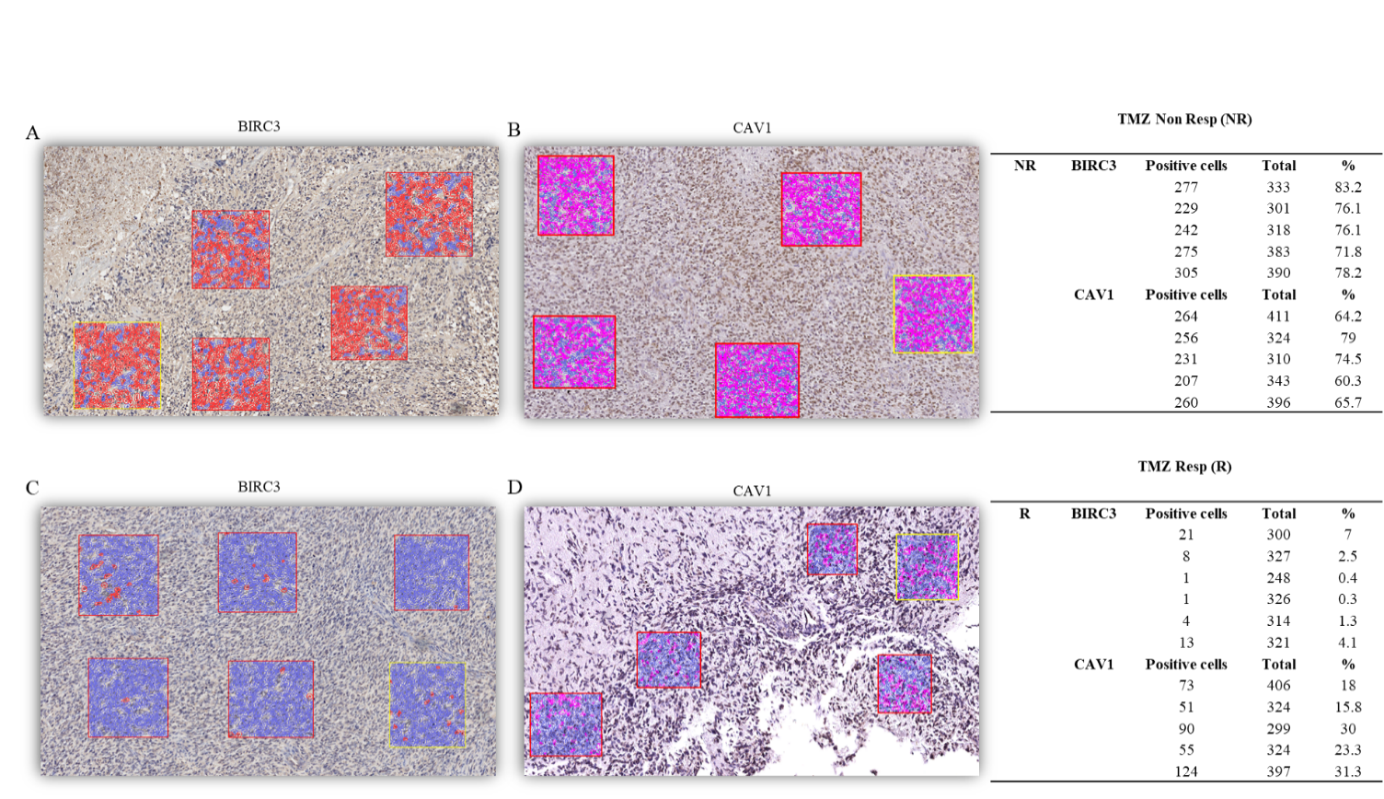


**Figure S3.** Multivariate Cox analyses demonstrating the independent prognostic value of *BIRC3* and *CAV1*. Forest plots show hazard ratios (HRs) for overall survival (OS). The analyses were designed to evaluate whether *BIRC3* and *CAV1* act as independent prognostic biomarkers, both in relation to *MGMT* promoter methylation and to each other. Models were tested across three TCGA datasets: RNA-seq (panels A–C), Agilent-4502A (D–F), and Affymetrix HG-U133A (G–I). For each dataset, the panels display: *BIRC3* + *MGMT* (A, D, G), *CAV1* + *MGMT* (B, E, H), *BIRC3* + *CAV1* (C, F, I). Patients were stratified into high and low expression groups using optimal cutoffs. HRs are shown with 95% confidence intervals.

**
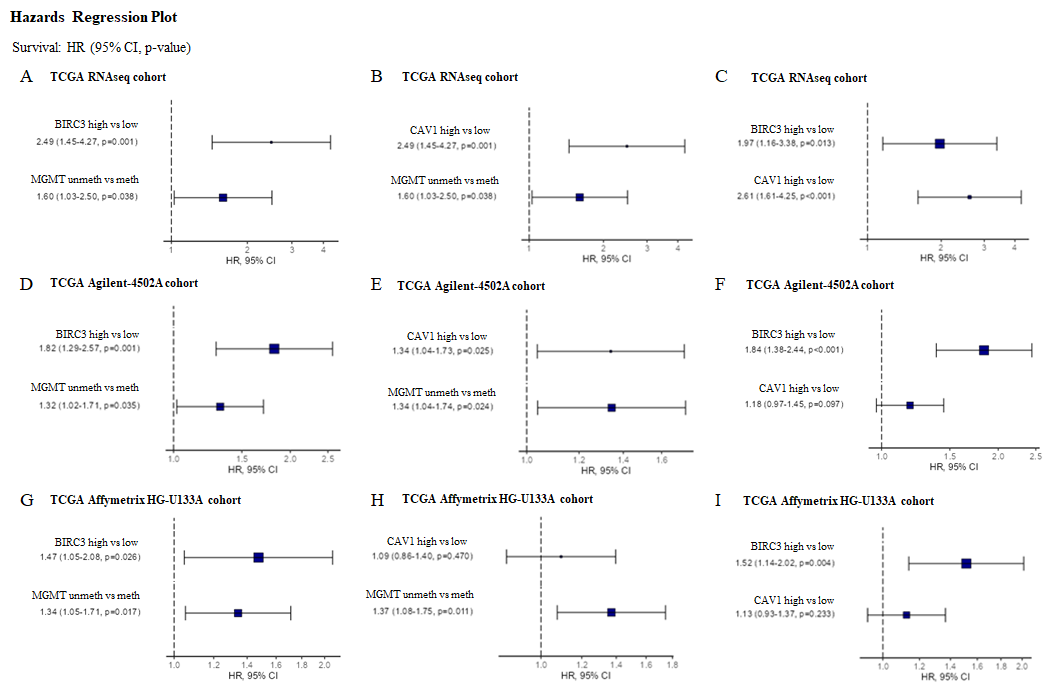
**

**Figure S4**. Non-linear and conditional prognostic effects of *BIRC3* and *CAV1* expression. (A) Cox spline model for continuous *BIRC3* expression (four knots), showing a predominantly linear association with the hazard ratio. (B) Cox spline model for *CAV1* (four knots), revealing a significant U-shaped relationship with risk (p for non-linearity = 6.8 × 10⁻⁴). (C) Additive spline model showing the hazard ratio for BIRC3 (x-axis) conditioned on three representative percentiles of CAV1 expression: 10th (red), 50th (green), and 90th (blue). Dashed vertical lines indicate the 10th and 90th percentiles of BIRC3 expression.


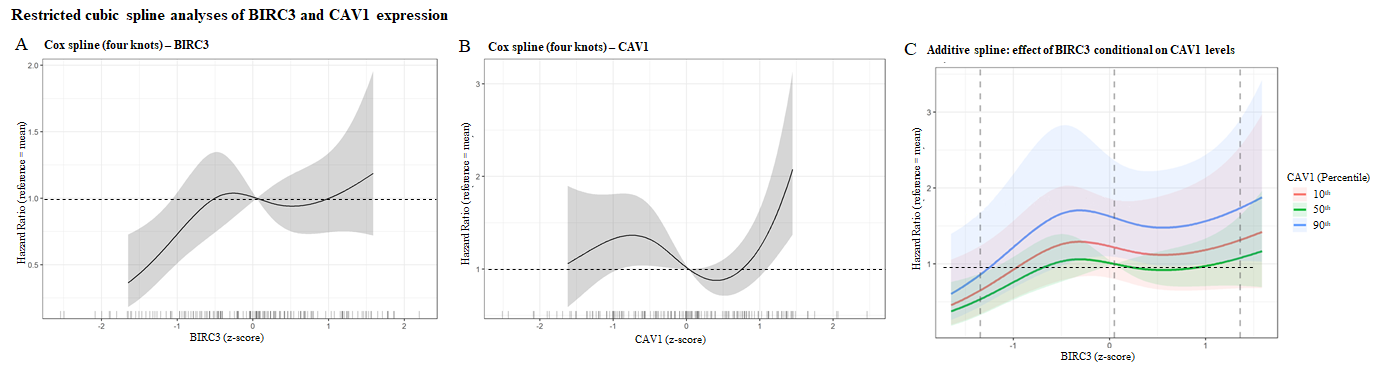


**Figure S5**. Forest plots of *BIRC3* and *CAV1* expression in discovery (RNA-seq) and validation (Agilent-4502A) datasets. Multivariable Cox regression analyses were adjusted for *MGMT* promoter methylation status. Hazard ratios (HRs) and 95% confidence intervals (CIs) are shown for “high” versus “low” expression groups, defined by a pre-specified rule (high: z > 0; low: z ≤ 0). The analysis includes individual gene expression as continuous variables (“value”), percentile-transformed variables (“percentile”), and a linear predictor (LP) combining *BIRC3* and *CAV1*.


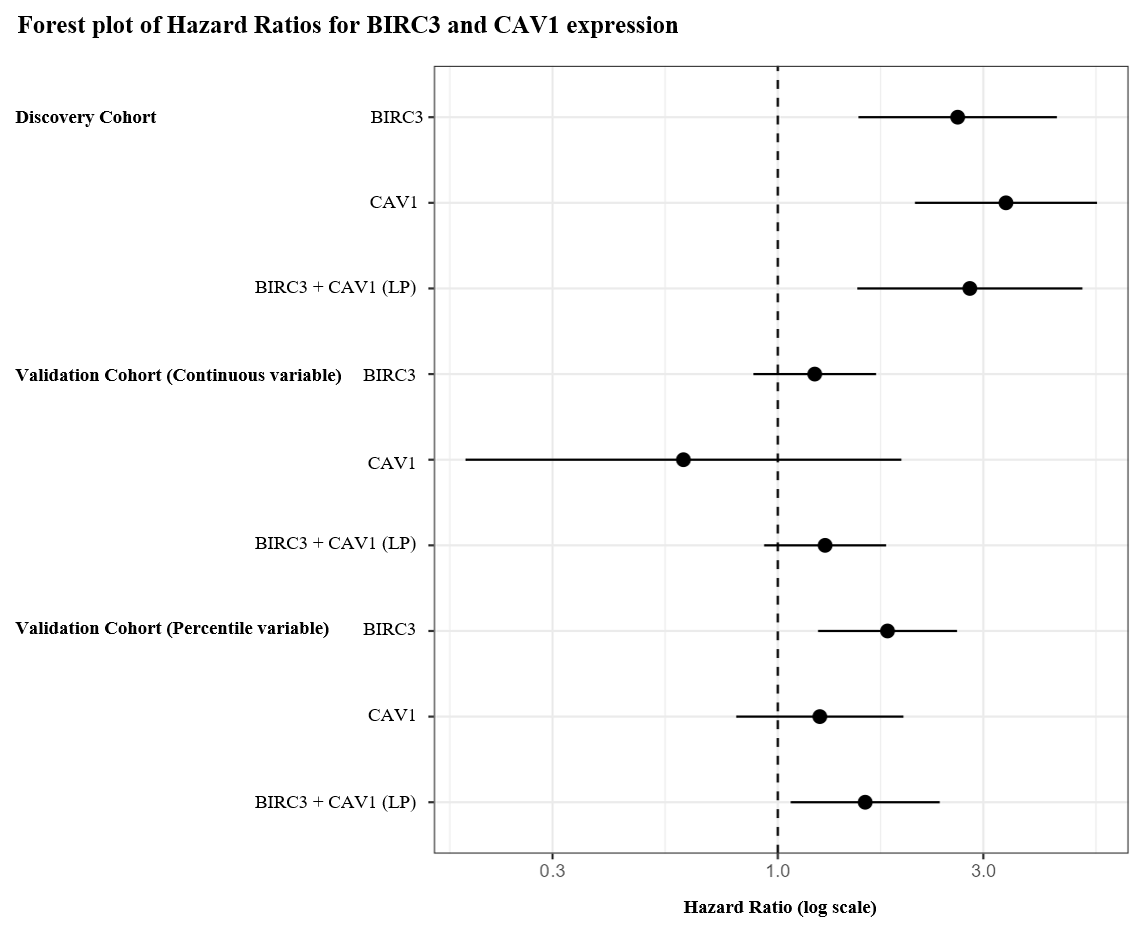


**Figure S6**. Transcriptional modulation of apoptotic markers in U87 cells. RT-qPCR analysis of BAX, BCL2, and CASP3 mRNA levels in U87 cells transfected with empty vector, BIRC3, CAV1, or BIRC3/CAV1 constructs, followed by treatment with TMZ (100 µM) or vehicle (DMSO) for 72 hours. Data are normalized to β-actin and expressed as fold change relative to Vector/DMSO controls. Bars represent mean ± SD of three independent biological replicates (P < 0.05 vs. respective DMSO control).

**
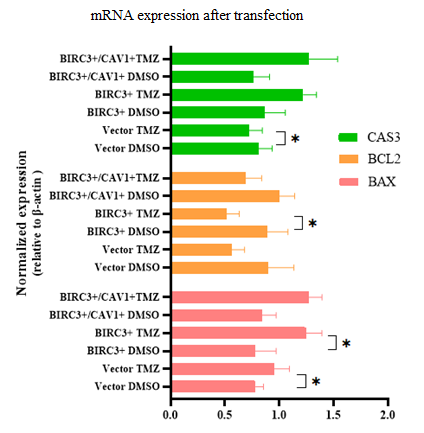
**

**Table S1**. Twelve-month survival probabilities (S₁₂) and cumulative risk of death (Risk₁₂) in TCGA RNA-seq GBM patients stratified by *BIRC3* and *CAV1* expression. Kaplan–Meier estimates (S₁₂ KM, Risk₁₂ KM) and Cox model–predicted probabilities (S₁₂ Cox, Risk₁₂ Cox) are shown.

| **Group** | **N** | **S_12_ KM** | **Risk_12_ KM** | **S_12_ COX** | **Risk_12_ COX** |
| --- | --- | --- | --- | --- | --- |
| Low *BIRC3* + Low *CAV1* | 31 | 0.828 | 0.172 | 0.814 | 0.186 |
| High *BIRC3* + Low *CAV1* | 100 | 0.602 | 0.398 | 0.607 | 0.393 |
| Low *BIRC3* + High *CAV1* | 0 | NA | NA | NA | NA |
| High *BIRC3* + High *CAV1* | 24 | 0.226 | 0.774 | 0.221 | 0.779 |

**Table S2**. Hazard ratios (HR), lower and upper confidence limits (LCL, UCL), and p-values for *BIRC3*, *CAV1*, and combined *BIRC3*/*CAV1* contrasts (90th vs 10th) in the TCGA RNA-seq cohort.

| **Marker** | **HR** | **LCL** | **UCL** | **p** |
| --- | --- | --- | --- | --- |
| BIRC3: 90th vs 10th | 2.142976 | 1.253011 | 3.665049 | 0.005374 |
| CAV1: 90th vs 10th | 1.454764 | 0.915161 | 2.31253 | 0.11295 |
| BIRC3 + CAV1: (90th,90th) vs (10th,10th) | 2.667502 | 1.427201 | 4.985677 | 0.002107 |

**Table S3**. Estimated survival probabilities (S₁₂, S₂₄) and cumulative risks (Risk₁₂, Risk₂₄) at selected *BIRC3* and *CAV1* percentiles from the additive Cox spline model. Columns LCL and UCL indicate the 95% confidence limits of the estimated survival probabilities S₁₂ and S₂₄.

| **Label** | **BIRC3 (z)** | **CAV1 (z)** | **S12** | **Risk12** | **LCL12** | **UCL12** | **S24** | **Risk24** | **LCL24** | **UCL24** |
| --- | --- | --- | --- | --- | --- | --- | --- | --- | --- | --- |
| BIRC3 10th / CAV1 10th | -1.347 | -1.281 | 0.735 | 0.265 | 0.622 | 0.868 | 0.368 | 0.632 | 0.222 | 0.609 |
| BIRC3 50th / CAV1 10th | 0.049 | -1.281 | 0.562 | 0.438 | 0.442 | 0.714 | 0.154 | 0.846 | 0.075 | 0.314 |
| BIRC3 90th / CAV1 10th | 1.359 | -1.281 | 0.537 | 0.463 | 0.377 | 0.763 | 0.132 | 0.868 | 0.045 | 0.39 |
| BIRC3 10th / CAV1 50th | -1.347 | 0.03 | 0.777 | 0.223 | 0.682 | 0.885 | 0.44 | 0.56 | 0.297 | 0.651 |
| BIRC3 50th / CAV1 50th | 0.049 | 0.03 | 0.623 | 0.377 | 0.521 | 0.745 | 0.215 | 0.785 | 0.127 | 0.363 |
| BIRC3 90th / CAV1 50th | 1.359 | 0.03 | 0.6 | 0.4 | 0.478 | 0.753 | 0.19 | 0.81 | 0.097 | 0.374 |
| BIRC3 10th / CAV1 90th | -1.347 | 1.32 | 0.666 | 0.334 | 0.524 | 0.846 | 0.267 | 0.733 | 0.123 | 0.58 |
| BIRC3 50th / CAV1 90th | 0.049 | 1.32 | 0.467 | 0.533 | 0.342 | 0.638 | 0.084 | 0.916 | 0.03 | 0.24 |
| BIRC3 90th / CAV1 90th | 1.359 | 1.32 | 0.44 | 0.56 | 0.315 | 0.614 | 0.069 | 0.931 | 0.023 | 0.209 |

**Table S4**. Densitometric quantification and Stain-Free normalization of Western blot data. The table reports the raw adjusted volume (Adj. Volume (Int)), the total protein load correction factor (Stain Free Nor), and the final normalized volume (Norm. Vol.) for BIRC3 and CAV1 protein levels across three independent biological replicates (Blots A-C). These data correspond to the single-transfected U87 cells (Vector, BIRC3+, and CAV1+) presented in Figure 5B. The final normalized volume, utilized for the scatter plots in the main text, was obtained by dividing the target protein adjusted volume by its corresponding Stain-Free normalization factor.

| **CAV1 blot A** | **Adj. Volume (Int)** | **Stain Free Norm. Factor** | **Norm. Vol. (Int)** |
| --- | --- | --- | --- |
| Vector | 165574113 | 0,768 | 254344270 |
| BIRC+ | 181836097 | 0,917 | 333556000 |
| CAV1+ | 228944780 | 1,000 | 457889560 |
| **CAV1 blot B** |  |  |  |
| Vector | 251752022 | 1,000 | 251752022 |
| BIRC+ | 280767900 | 1,192 | 334765562 |
| CAV1+ | 324609810 | 1,315 | 426938836 |
| **CAV1 blot C** |  |  |  |
| Vector | 257390931 | 1,000 | 257390931 |
| BIRC+ | 280199574 | 1,192 | 334087934 |
| CAV1+ | 317476888 | 1,315 | 417557353 |
| **BIRC3 blot A** |  |  |  |
| Vector | 134124286 | 1,000 | 134124286 |
| BIRC+ | 185471339 | 1,192 | 221141437 |
| CAV1+ | 63633970 | 1,315 | 83693752 |
| **BIRC3 blot B** |  |  |  |
| Vector | 126705869 | 1,000 | 126705869 |
| BIRC+ | 184918470 | 1,192 | 220482240 |
| CAV1+ | 58662750 | 1,315 | 77155420 |
| **BIRC3 blot C** |  |  |  |
| Vector | 120002982 | 1,000 | 120002982 |
| BIRC+ | 172125954 | 1,192 | 205229450 |
| CAV1+ | 72779490 | 1,315 | 95722278 |

**Table S5.** Densitometric quantification and Stain-Free normalization of Western blot data (T98G cells). The table reports the raw adjusted volume (Adj. Volume (Int)), the total protein load correction factor (Stain Free Nor), and the final normalized volume (Norm. Vol.) for BIRC3 and Cleaved Caspase-3 (CC3) protein levels across three independent biological replicates (Blots A-C). These data correspond to the T98G cells treated with DMSO, TMZ, AZD, or the combination (TMZ+AZD) presented in Figure 6H. The final normalized volume was obtained by dividing the target protein adjusted volume by its corresponding Stain-Free normalization factor.

| **BIRC3 blot A** | **Adj. Volume (Int)** | **Stain Free Norm. Factor** | **Norm. Vol. (Int)** |
| --- | --- | --- | --- |
| DMSO | 17189886 | 1,000 | 17189886 |
| TMZ | 88584378 | 1,106 | 98029870 |
| AZD | 83899450 | 1,155 | 96978241 |
| TMZ+AZD | 26636586 | 1,190 | 31716655 |
| **BIRC3 blot B** |  |  |  |
| DMSO | 19956249 | 1,000 | 19956249 |
| TMZ | 93429083 | 1,049 | 98060202 |
| AZD | 84451248 | 1,178 | 99544625 |
| TMZ+AZD | 30470497 | 1,218 | 37118638 |
| **BIRC3 blot C** |  |  |  |
| DMSO | 20781459 | 1,000 | 20781433 |
| TMZ | 83067961 | 1,049 | 87185388 |
| AZD | 68710771 | 1,178 | 80990862 |
| TMZ+AZD | 38269031 | 1,218 | 46618621 |
| **CC3 blot A** |  |  |  |
| DMSO | 9848160 | 1,000 | 9848160 |
| TMZ | 102270870 | 1,049 | 107340262 |
| AZD | 55808935 | 1,178 | 65783273 |
| TMZ+AZD | 32434910 | 1,218 | 39511653 |
| **CC3 blot B** |  |  |  |
| DMSO | 6651548 | 1,000 | 6651548 |
| TMZ | 113322095 | 1,056 | 119772078 |
| AZD | 67980524 | 1,191 | 80970668 |
| TMZ+AZD | 34805388 | 1,226 | 42703331 |
| **CC3blot C** |  |  |  |
| DMSO | 12776014 | 1,000 | 12776014 |
| TMZ | 115661971 | 1,051 | 121563062 |
| AZD | 71213422 | 1,181 | 84127847 |
| TMZ+AZD | 36836355 | 1,219 | 44933073 |

**Supplementary Data Legends**

**Supplementary Data 1**: Transcriptomic analysis of TMZ-resistant vs. responder GBM explants. Related to Figure 1. Complete results of differential gene expression analysis (RNA-seq) on the expanded cohort of 33 GBM samples, comparing Non-Responder (Non-Resp, n = 17) and Responder (Resp, n = 16) samples to TMZ. The table includes the genes analyzed, log2 fold change values, p-values, and adjusted p-values (padj).

**Supplementary Data 2**: Data from the French validation cohort. Related to Figure 6. Clinical and expression dataset for the French validation cohort (n = 228) of IDH-wildtype GBM patients treated with the Stupp protocol. The table includes clinical data (Overall Survival, Progression-Free Survival, MGMT methylation status) and corresponding expression data for BIRC3 and CAV1 (RNA-seq).

**Supplementary Data 3**: Data from the Italian validation cohort. Related to Figure 7. Clinical and expression dataset for the Italian validation cohort (n = 68) of patients with IDH-wildtype GBM (48 primary and 20 recurrent). The table includes clinical data (Overall Survival, Progression-Free Survival, MGMT methylation status) and corresponding expression data for BIRC3 and CAV1 (RT-qPCR).
