# Supplementary material for: BIRC3/CAV1 co-expression drives GBM aggressiveness as a prognostic signature and therapeutic vulnerability
Source: Cell Death Discov. 2026 Apr 14;12:232. doi: 10.1038/s41420-026-03112-z (PMC13183958; doi:10.1038/s41420-026-03112-z)
Supplement: Supplementary file 4 — Supplementary Data 3 [file 41420_2026_3112_MOESM4_ESM.pdf]

GB code	Sex	Age	P/R	MGMT	BIRC3	BIRC3 cutof
GB 34	m	56	P	Meth	0.07949079	0
GB 35	f	43	P	Meth	9.75	1
GB 36	m	38	P	Meth	0.35492259	1
GB 37	f	49	P	Meth	0.02795	0
GB 38	m	47	P	Unmeth	0.35132742	1
GB 39	m	82	P	Meth	0.10542803	0
GB 40	m	73	P	Meth	0.09371072	0
GB 41	f	52	P	Meth	0.0647	0
GB 42	f	65	P	Unmeth	0.0818	0
GB 43	m	74	P	Meth	0.34135577	1
GB 44	f	70	P	Unmeth	0.10402	0
GB 45	m	57	P	Meth	0.00494258	0
GB 46	m	72	R	Unmeth	0.09383452	0
GB 47	m	30	P	Unmeth	0.14193039	0
GB 48	f	46	P	Meth	0.05055	0
GB 49	m	71	P	Meth	0.07042106	0
GB 50	m	69	R	Meth	nd	
GB 51	m	64	P	Unmeth	0.19701394	1
GB 52	m	76	P	Meth	1.20859468	1
GB 53	f	73	P	nd	10	1
GB 54	m	56	R	Unmeth	9.77371206	1
GB 55	f	73	P	Unmeth	0.66178241	1
GB 56	m	70	P	Meth	1.12530201	1
GB 57	m	82	P	nd	0.03805	0
GB 58	m	55	R	Unmeth	0.78474374	1
GB 59	m	77	R	Meth	0.41500171	1
GB 60	m	52	R	Unmeth	4.2823509	1
GB 61	f	55	P	Unmeth	2.44939252	1
GB 62	m	75	P	Meth	0.35630197	1
GB 63	m	60	R	Meth	0.0163857	0
GB 64	m	72	R	Meth	1.79	1
GB 65	m	56	P	Meth	6.0579697	1
GB 66	m	82	P	Meth	0.47038028	1
GB 67	f	74	P	Meth	0.70716386	1
GB 68	m	72	R	Meth	0.67258111	1
GB 69	m	30	P	Unmeth	2.08037325	1
GB 70	m	69	P	Unmeth	0.87646076	1
GB 71	m	77	P	Meth	0.02158607	0
GB 72	m	68	R	Unmeth	0.16683361	1
GB 73	m	68	R	Unmeth	0.19631321	1
GB 74	f	74	R	nd	0.03768631	0
GB 75	m	72	R	Meth	0.04224395	0
GB 76	m	49	P	Meth	0.0114795	0
GB 77	m	68	P	Unmeth	0.11791	0
GB 78	m	76	P	Meth	0.07810046	0
GB 79	m	52	P	Meth	nd	
GB 80	f	56	R	Unmeth	0.31175241	1

GB 81	f	71	P	Meth	1.37923629	1
GB 82	f	67	P	Unmeth	0.05517508	0
GB 83	f	78	P	Meth	0.0155	0
GB 84	m	75	P	Meth	0.04544079	0
GB 85	f	36	P	Unmeth	0.043448	0
GB 86	m	64	P	Meth	0.02283	0
GB 87	m	76	P	Meth	0.05373	0
GB 88	m	74	R	Meth	0.52	1
GB 89	m	58	P	Unmeth	0.03959	0
GB 90	f	52	R	Unmeth	0.20208	1
GB 91	m	76	R	Meth	0.153	0
GB 92	m	55	R	nd	0.0413	0
GB 93	m	77	R	Meth	0.12447	0
GB 94	f	61	R	Meth	0.03017104	0
GB 95	m	82	P	Meth	0.11251213	0
GB 96	m	79	P	Meth	0.77989303	1
GB 97	nd	nd	P	Meth	0.45944739	1
GB 98	m	83	P	Meth	0.04168	0
GB 99	f	82	P	Meth	0.29479	1
GB 100	m	73	P	Meth	0.19	1
GB 101	m	72	P	Unmeth	0.60693449	1

**CAV1**	**CAV1 cutoff**	**PFS months**	**Progression (**	**OS months**	**Death (1/0)**
0.06646648	0	24	0	43	0
0.42875546	1	28	0	34	0
0.64742363	1	12	0	16	0
0.08399828	0	11	0	20	0
0.05532752	0	5	0	11	1
0.37266266	1	12	0	17	1
0.1538452	1	9	0	16	1
nd	nd	1	0	2	1
nd	nd	16	0	17	1
0.22499349	1	10	1	27	0
nd	nd	16	1	24	0
0.01530437	0	10	1	17	1
0.01876707	0	7	1	23	1
0.04333182	0	7	1	15	1
nd	nd	14	1	14	1
0.02808836	0	12	1	22	1
0.0760785	0	4	1	16	1
0.09833957	1	4	1	16	1
0.03690388	0	7	1	10	1
nd	nd	12	1	17	1
0.09707868	1	17	1	24	1
0.01092787	0	12	1	19	1
0.25256037	1	8	1	10	1
nd	nd	9	1	12	1
0.06078207	0	4	1	16	1
0.03523552	0	39	1	43	1
0.21441844	1	7	1	15	1
0.86316855	1	16	1	22	1
0.02599692	0	15	1	20	1
0.02980933	0	17	1	22	1
0.069	0	11	1	22	1
6.0579697	1	3	1	5	1
1.44852991	1	2	1	2	1
0.86203568	1	1	1	1	1
0.05230519	0	8	1	14	1
0.15092606	1	11	1	15	1
1.74286028	1	7	1	12	1
0.05948901	0	7	1	29	1
1.02051537	1	9	1	13	1
0.11110146	1	9	1	13	1
0.05923738	0	12	1	19	1
0.09491223	1	11	1	12	1
0.05199855	0	13	1	19	1
1.64253257	1	6	1	8	1
0.42134501	1	23	1	29	1
0.02	0	1	1	1	1
0.15440713	1	2	1	11	1

0.1	1	5	1	5	1
0.04938337	0	18	1	23	1
nd	nd	1	1	1	1
0.10144132	1	3	1	6	1
nd	nd	5	1	7	1
nd	nd	7	1	8	1
nd	nd	13	1	19	1
0.07	0	29	1	44	1
nd	nd	4	1	8	1
nd	nd	4	1	11	1
nd	nd	15	1	26	1
nd	nd	61	1	81	1
nd	nd	7	1	29	1
0.53758877	1	28	1	nd	nd
0.00128633	0	nd	nd	3	1
0.11570102	1	nd	nd	4	1
0.27098694	1	nd	nd	4	1
0.21	1	nd	nd	8	1
0.17	1	nd	nd	9	1
0.03	0	nd	nd	9	1
0.17190658	1	nd	nd	1	1
