# Supplementary figures and images for: BIRC3/CAV1 co-expression drives GBM aggressiveness as a prognostic signature and therapeutic vulnerability
Source: Cell Death Discov. 2026 Apr 14;12:232. doi: 10.1038/s41420-026-03112-z (PMC13183958; doi:10.1038/s41420-026-03112-z)

BIRC3 3130

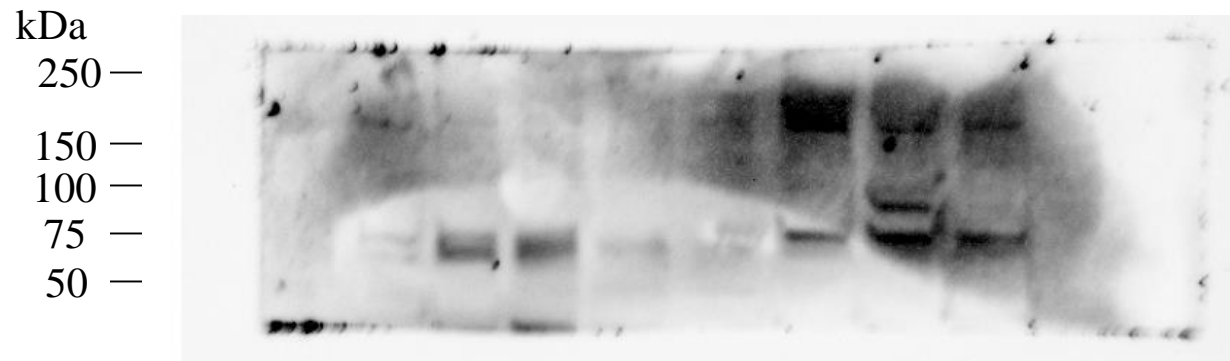

CC3

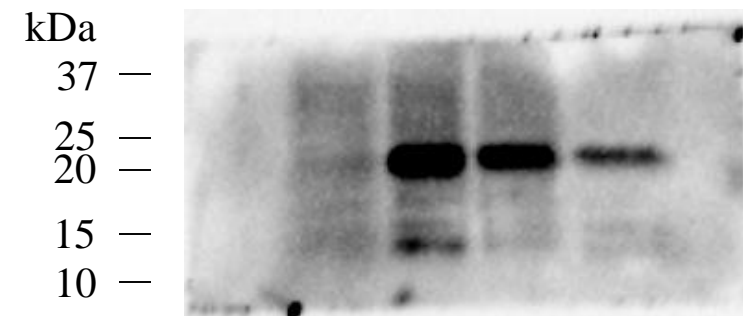

CAV1

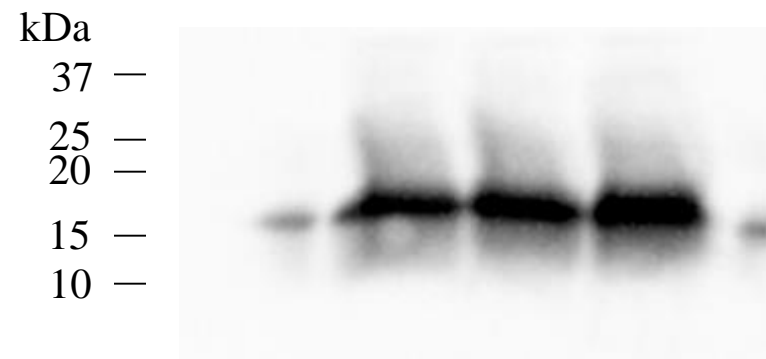

BIRC3 HP002317

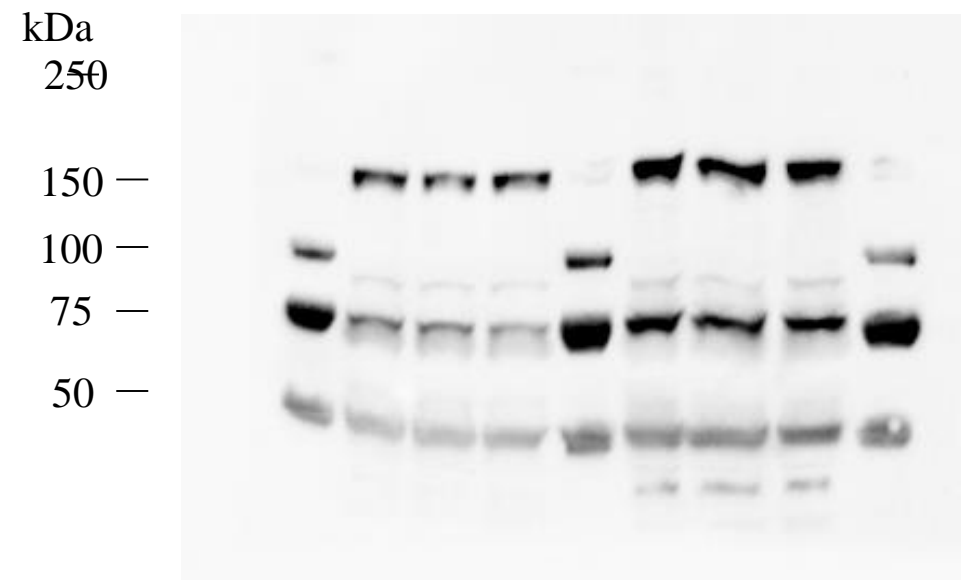

Supplement: Supplementary file 5 — Original Blots [file 41420_2026_3112_MOESM5_ESM.pdf]
